# The general practitioners perspective regarding registration of persistent somatic symptoms in primary care: a survey

**DOI:** 10.1186/s12875-021-01525-6

**Published:** 2021-09-11

**Authors:** Willeke M. Kitselaar, Rosalie van der Vaart, Madelon van Tilborg-den Boeft, Hedwig M. M. Vos, Mattijs E. Numans, Andrea W. M. Evers

**Affiliations:** 1 Health, Medical and Neuropsychology Department, Leiden University, Faculty of Social and Behavioral Sciences, Leiden, the Netherlands; 2 Public Health and Primary Care Department / LUMC-Campus Den Haag, Leiden University Medical Center, The Hague, the Netherlands

**Keywords:** Clinical Coding, Electronic Health Records, General Practitioners, Medically Unexplained Symptoms, Persistent Somatic Symptoms, Primary Health Care, Classification of Disease

## Abstract

**Background:**

Persistent somatic symptoms (PSS) are common in primary care and often accompanied by an increasing disease burden for both the patient and healthcare. In medical practice, PSS is historically considered a diagnosis by exclusion or primarily seen as psychological. Besides, registration of PSS in electronic health records (EHR) is unambiguous and possibly does not reflect classification adequately. The present study explores how general practitioners (GPs) currently register PSS, and their view regarding the need for improvements in classification, registration, and consultations.

**Method:**

Dutch GPs were invited by email to participate in a national cross-sectional online survey. The survey addressed ICPC-codes used by GPs to register PSS, PSS-related terminology added to free text areas, usage of PSS-related syndrome codes, and GPs’ need for improvement of PSS classification, registration and care.

**Results:**

GPs (n = 259) were most likely to use codes specific to the symptom presented (89.3%). PSS-related terminology in free-text areas was used sparsely. PSS-related syndrome codes were reportedly used by 91.5% of GPs, but this was primarily the case for the code for irritable bowel syndrome. The ambiguous registration of PSS is reported as problematic by 47.9% of GPs. Over 56.7% of GPs reported needing additional training, tools or other support for PSS classification and consultation. GPs also reported needing other referral options and better guidelines.

**Conclusions:**

Registration of PSS in primary care is currently ambiguous. Approximately half of GPs felt a need for more options for registration of PSS and reported a need for further support. In order to improve classification, registration and care for patients with PSS, there is a need for a more appropriate coding scheme and additional training.

**Supplementary Information:**

The online version contains supplementary material available at 10.1186/s12875-021-01525-6.

## Introduction

Up to 50% of primary care visits in Western societies are related to symptoms that cannot be fully explained by well-known biomedical pathology [1–4]. While most of these symptoms are self-limiting, 2.5–10% of cases persist without clear medical explanation [5–9]. These persistent somatic symptoms (PSS) are accompanied by increasing disease burden for both the patient and the healthcare system [10]. Differentiation from well-known chronic medical conditions and classification of symptoms as PSS is challenging [11]. Challenges arise from similarities between symptoms of PSS and other conditions, possible co-existence with a well-documented medical disorder, the heterogeneity of symptoms, lack of universal guidelines and the lack of biomarkers [3, 12–14]. Delayed identification of PSS impedes early management of symptoms, which in turn can result in inappropriate healthcare utilization and high costs [15–17]. Additionally, it may hinder reusability of electronic health records (EHR) for research, quality monitoring and proactive population health management [17–20].

Across medical and psychological specialties, a variety of terminology and aetiology is reflected in different concepts of PSS. While some countries have specific guidelines for PSS, widely accepted guidelines for classifying (and treating) PSS are missing [3]. PSS is currently diagnosed as either a somatic disease or a mental disorder, since diagnostic classifications are inherently dualistic in nature [21]. In the medical field, patients may be classified under umbrella terms such as ‘medically unexplained physical symptoms’ (MUPS), ‘functional somatic symptoms’, and ‘somatically fixed’ [9, 22], which indicate a negative symptomology – i.e. a lack of medical pathology [23]. PSS may also be classified as syndromes such as irritable bowel syndrome (IBS), chronic fatigue syndrome (CFS) or fibromyalgia (FM). Ongoing debate about terminology has redirected the most recent versions of the diagnostic and statistical manual of mental disorders (DSM-5) [24] and the international classification of disease (ICD-11) [25] towards no longer requiring the explicit exclusion of any underlying medical condition (this only applies to PSS-related classifications in the mental health chapter). Both focus on positive symptomology, such as maladaptive cognitions, emotions and/or behaviours related to the somatic symptoms [3, 13], as described in the DSM-5 as the so-called B-criteria of somatic symptom disorder (SSD) [24, 26]. Still, consensus on labelling and addressing these symptoms is limited. In this paper, the term ‘persistent somatic symptoms’ (PSS) is used since the descriptive nature of the term transcends the problem of dualism. Moreover, recent research has found that this term is generally preferred over other terms [27].

In the Dutch health care system, as well as in many (Western) countries, the GP serves as a gatekeeper for health care in general. The classification of symptoms and illnesses in EHRs by Dutch GPs is based on the International Classification of Primary Care (ICPC) system [28]. Since medical practice historically operates according to mind–body dualism, physicians are required to locate complaints either in the body or the mind [21]. Accordingly, most symptoms and disorders – physical and psychological – have a domain specific diagnostic code in the ICPC. Nonetheless, the current ICPC lacks a specific and clearly defined code for PSS and the ICPC system instructs to register symptoms not fulfilling the criteria for a diagnosis on a symptom level [28, 29]. Arguably, registration of cases with PSS is less straight forward due to the multi-domain nature of PSS even though the ICPC does contain a chapter with multi-domain codes (A-chapter). Nonetheless, there are international codes available for some PSS-related syndromes (such as, IBS), and the Dutch ICPC also contains codes for FM and CFS [28].

While several studies have documented ample diagnostic variation regarding patients with PSS in general practice [30, 31], it is not well documented which codes or other methods GPs use for registration of PSS and if they find their current approach to registration satisfactory. The primary aim of the present study was, therefore, to explore how GPs currently register PSS. The secondary aim was to gauge GPs’ perspective on their needs to improve classification, registration and care for PSS.

## Methods

### Study design

A cross-sectional online survey was developed to reach our aims. The survey questions were developed in collaboration with experts in general practice, medical psychology and PSS. The survey was set up in Qualtrics [32]. This paper focuses on GPs’ registration behaviour and needs, using the STROBE cross-sectional reporting guidelines [33]. Prior to distribution, the survey was pilot tested among four GPs and modified based on their feedback. Informed consent was included at the start of the survey. The ethics committee of Leiden University Medical Centre supplied a waiver of ethical approval (C1O8.045/DJ/gk).

### Procedure

The survey was sent out via e-mail between June and September 2018 to mailing lists of Dutch GPs who consented to be approached anonymously for research purposes, and to email addresses obtained through an overarching Dutch healthcare website (www.zorgkaartnederland.nl). This method ensured optimal distribution over all regions of the Netherlands in order to obtain a representative and generalizable sample. Reminders were sent two weeks after first distribution. Ten gift cards of 25 euro were allotted to GPs who participated and provided us with their email address. The email addresses were not linked to the survey responses.

### Measures

To adhere to the term currently used in guidelines for PSS-related complaints, the Dutch term for MUPS (‘SOLK’) was used to indicate PSS in the survey. Somatisch onvoldoende verklaarde lichamelijke klachten (SOLK) is literally translated as somatic insufficiently explained physical complaints. In the introduction of the survey, a description of the definition of SOLK was presented: ‘We speak of SOLK when regular medical care cannot find an adequate explanation for the complaints with which the patient presents him/herself. Patients with a well-known somatic condition can also have SOLK, either presenting with a totally different complaint or presenting with more severe complaints than is expected.’ Distinction and explanation about self-limiting and persistent symptoms were provided. To address conceptual differences between GPs regarding PSS and to ensure that both the medical and psychological domain of PSS was captured, separate questions were added which specifically addressed PSS in patients with a(n explained) chronic medical condition (i.e., ‘patients presenting with more or more severe symptoms than you would expect’) and/or the B-criteria of SSD (i.e., ‘patients who have maladaptive cognitions, emotions and/or behaviours related to the somatic symptoms’). At the start of the survey, GPs were asked to fill in non-identifying demographic questions. All questions required at least one response to continue to the next question, except comment sections. Below you find a description of the survey questions (for an exact outline of the survey, see additional file 1).

*Primary aim (‘registration of PSS’)*– The following four items were constructed to reach the primary aim: (1) First a description of a hypothetical patient was given as follows: ‘Imagine a patient visiting your office who has consulted you frequently in the previous 6 months with the same or differing complaints. Extensive research has excluded a medical explanation for the complaint(s). For each complaint presented, choose the ICPC-code which you would use most often. You can choose a maximum of three ICPC-codes per complaint.’ Then followed 4 complaints on different pages: bowel problems, fatigue, neck and back pain, and shortness of breath. A drop-down menu contained all codes related to the complaint in the thesaurus menu from ICPC-online [34], which reflects the presentation in GPs’ EHR. This list was supplemented with suitable codes based on a PSS-expert panel of GPs (see additional file 2 for the full list of ICPC-codes from which GPs could choose). The four separate complaints were offered in random order to minimize bias due to presentation order. Next, (2) GPs were asked whether they use the PSS-related syndrome codes A04.01 (CFS), D93 (IBS) and/or L18.01 (FM). Respondents selected one or more of five options: ‘Yes, I diagnose the syndromes myself sometimes’, ‘Yes, I use this code when the syndrome is diagnosed by a medical specialist’, ‘No, I think these complaints should be reported on a symptom level’, ‘No, I am not convinced these are distinguishable syndromes’, and ‘Other, namely…’ (with an additional comment section). This question was added to the survey in a later stage and was therefore only presented to 73% (n = 189) of the GPs.

Subsequently, (3) GPs were asked whether they mention PSS-related terminology in the (3a) episode name or (3b) free text area (two 4-point scale items (ranging from ‘never’ to ‘always’)). Lastly, (4) a description of a hypothetical consultation with a patient with a diagnosed medical condition was given, whereby the patient presented with specific cognitive, emotional and behavioural problems (conforming to the B-criteria of SSD) [24]. GPs were asked if they would mention this in their EHR (yes/no, and a comment section).

*Secondary aim (‘GPs needs’) –* The following four items were constructed to reach the secondary aim: First, (1) GPs were asked if the lack of an unambiguous way of classifying or coding PSS was problematic for them (yes/no, and a comment section). Next, (2) GPs were asked if they had a need for a code which captures the specific cognitions, emotions and behaviour conforming to the B-criteria of SSD (yes/no, and a comment section). Subsequently, an open-ended question was presented where GPs were asked (3) what they need to be able to improve registration and classification of PSS; and, in order to ensure not missing any needs, this was followed by three specific semi-open-ended questions – (4) if they have needs regarding training, (online) tools or other support, to improve consultations and classification of PSS (response options were: ‘no’ and ‘yes, namely…’).

### Data analysis

All results are based on descriptive statistics. Survey responses were summarized as is, using sample sizes and percentages, unless otherwise specified above. For the first hypothetical consultation, codes were first categorized into four groups: symptom-specific codes (e.g., A04-fatigue), general codes – i.e., non-specific codes (e.g., P28-limited function/disability(p)), somatization (P75-somatization disorder) and syndromes (A04.01-CFS, D93-IBS and L18.01-FM) (see additional file 2). The responses on the four single complaints were analysed both combined and as separate complaints. For the question regarding the use of PSS-related syndrome codes, the two ‘Yes, …’ answering options were combined and the two ‘No, …’ answering options were combined to construct total scores.

## Results

Of the approximately 12,000 active GPs in the Netherlands, an estimated 2,000 GPs were reached through our distribution method. In total, 259 GPs (13%) fully completed the survey, with exception to the fourth item (4) which was completed by 189 GPs. Table 1 displays the characteristics of the total sample. Of the GPs who filled out the survey, 60.2% were female, which reflects the current trend towards increasing numbers of female GPs in the Netherlands [35]. GPs from all regions in the Netherlands completed the survey. GPs years since graduation is reasonably evenly distributed over 5-year periods, varying between the smallest group of GPs graduating between 26 to 30 years since participating in the survey (8.5%) and the largest group of GPs graduating between 6 to 10 years before participation (17.8%).

**Table 1:** Characteristics of the 259 Dutch GPs participating in the study

General practitioners	n = 259 (%)
Gender (female)	156 (60.2)
Years since graduation	
< *5*	41 (15.8)
* 6—10*	46 (17.8)
* 11—15*	44 (14.0)
* 16—20*	39 (15.1)
* 21—25*	31 (12.0)
* 26—30*	22 (8.5)
> *30*	36 (13.9)
Location of practice	
* Urban (Randstad)*	81 (31.0)
* North*	62 (23.8)
* Middle*	102 (35.6)
* South*	23 (8.8)

### Registration of PSS

**Fig. 1:**
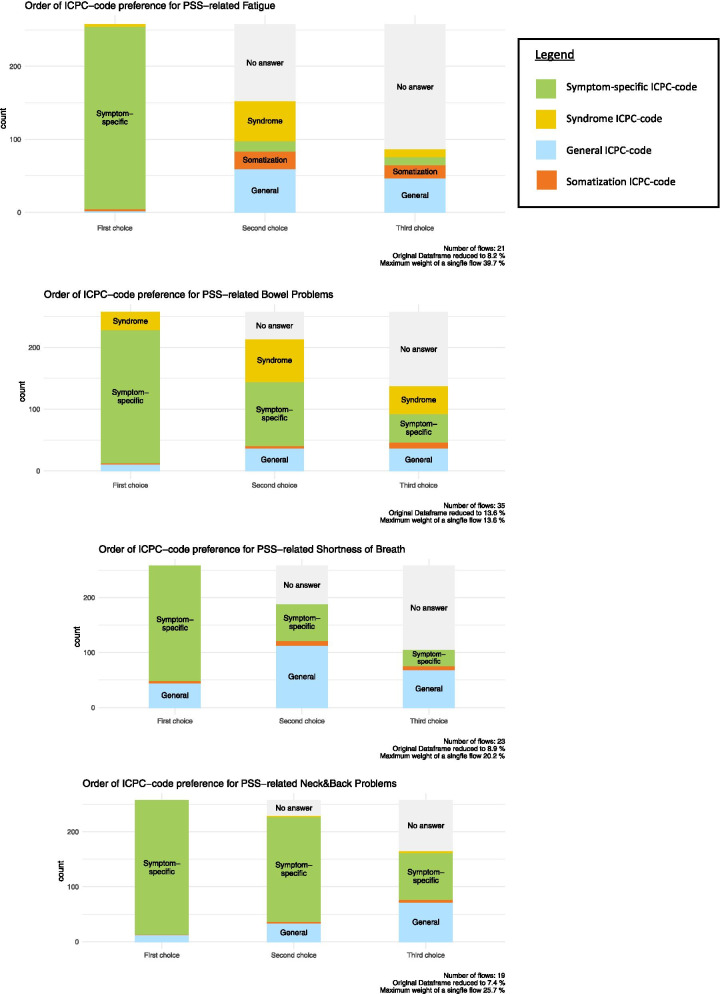
Visualizations of general practitioners’ order of choosing ICPC-codes for specified persistent somatic symptoms (PSS)

As shown in Fig. 1, GPs vary in their way of reporting PSS. Combining the preferred first choices of code for the four PSS case examples, the general trend indicates that GPs were most likely to register PSS on a symptom-specific level (89.3%). The frequency of choosing general codes increased from 6.9% as a first choice to 31.1% for the second and 45.5% for the third choice. The choice for ICPC code P75 (somatization disorder) increased from 1% as a first choice, to 5.1% and 8.0% for the second and third choice respectively. When presented with fatigue complaints, more than 35.7% chose to report the complaint with P75 (as a second or third choice). Looking at the number of times a syndrome code (IBS, CFS or FM) was generally chosen either as a first, second or third choice, 144 chose D93 (IBS) in case of bowel complaints, 69 GPs chose A04.01 (CFS) in case of fatigue complaints, and 6 chose L18.01 (FM) in case of neck and back pain. For a more detailed description of the choices of ICPC codes per presented symptom, see additional file 3.

Table 2 shows the reported likelihood that GPs mention PSS-related terminology and cognitions, emotions or behaviour related to PSS in their EHR. Some GPs mentioned in the comment section that a fear of stigmatization was the reason for avoiding PSS-related terms.

**Table 2:** GPs (n = 259) mentioning PSS-related terminology in their EHR

Do you mention PSS in the episode name?	n (%)
* never*	79 (30.5)
* occasionally*	146 (56.4)
* often*	28 (10.8)
* always*	6 (2.3)
**Do you mention PSS in the free-text area?**	
* never*	29 (11.2)
* occasionally*	156 (60.2)
* often*	64 (24.7)
* always*	10 (3.9)
**Do you mention components**^a^**of PSS in the free-text area?**	
* Yes*	204 (78.8)

^a^ Specific thoughts, feelings and behaviour conforming to the B-criteria of SSD

Of all GPs who answered the question regarding ICPC-codes for recognized PSS-syndromes (n = 189), 91.5% indicated that they use the codes for IBS (D93), CVS (A04.01) and FM (L18.01) (not shown in Table). The answering options given in the survey are depicted in Table 3. While 68.3% of GPs reported diagnosing the syndrome themselves, several GPs commented that this was only the case for IBS (which was also the case in most GPs who selected the answer ‘Other, namely…’).

**Table 3:** GPs (n = 189) use of PSS-related syndrome codes

**Do you use the PSS-syndrome ICPC codes?**^a,b^	n = 189
* Yes, I diagnose the syndromes myself sometimes*	129 (68.3)
* Yes, I use this code when the syndrome is diagnosed by a medical specialist*	74 (39.2)
* No, I think these complaints should be reported on a symptom level*	25 (12.2)
* No, I am not convinced these are distinguishable syndromes*	9 (4.8)
* Other, namely…*	19 (10.1)

^a^ A04.01 – CFS; D93 – IBS; L18.01—FM

^b^ Multiple answers possible per GP

### GPs needs

Table 4 shows the results relating to the second aim of this study. Approximately half of GPs (47.9%) reported that the lack of an unambiguous way of classifying or coding PSS is a problem for them. Many GPs commented that a specific code for PSS would be helpful, and some suggested that PSS-codes per tract or a specific code with different severity levels would be helpful. GPs commented requiring widely accepted guidelines in combination with a new PSS-code. Of those who did not see the lack of a specific PSS code as a problem (52.1%), many commented they sometimes describe PSS in the available free text area when registering the patient’s somatic complaint. Others felt there is still too much uncertainty regarding PSS to code it, felt unwilling to apply “that label” to a patient, or commented that registration at a symptom level was sufficient. Of all GPs, 32.8% reported that they would like to be able to express PSS-related components – i.e., specific thoughts, feelings and behaviour conforming to the B-criteria of SSD – in a code. Although some of these GPs commented that they found it difficult to specify the components. Additionally, GPs indicated a need for training (56.7%) and/or an – (online) classification and/or risk assessment – tool (58.3%) and/or other support (69.7%). Other PSS-related needs mentioned by GPs in the elective comment sections regarded clearer or more referral options, more time and financial compensation for consultations and better guidelines (although others explicitly mentioned that they found the current guidelines adequate).

**Table 4:** GPs’ needs for improving registration and classification of persistent somatic symptoms in EHR

There is no ICPC code for PSS; is this a problem for you?	n = 259 (%)
* Yes*	127 (47.9)
**Would you like to express components **^**a**^** of PSS in an ICPC code?**	**n = 259 (%)**
* Yes*	85 (32.8)
**Do you have a need for … to improve consultations/classification for PSS?**	**n = 254 (%)**
*…training… (yes)*	144 (56.7)
*…an (online) tool… (yes)*	148 (58.3)
*…other support… (yes)*	177 (69.7)

^a^ Specific thoughts, feelings and behaviour conforming to the B-criteria of SSD

## Discussion and conclusion

### Discussion

The results of this survey indicate that codes used for registration of PSS in primary care varies widely among GPs. PSS are primarily coded at a specific somatic symptom level and GPs often avoid using terminology related to PSS in their EHR. In addition, GPs indicate they us the codes for well-known PSS-syndromes as IBS, CFS, or FM, although IBS is coded more often than CFS and FM. Besides, the cognitive, emotional or behavioural components of PSS are sparsely reported in EHRs. Some GPs indicated that they have difficulties in specifying these components. Overall, half of GPs are unsatisfied with current registration options for PSS. Many GPs have a need for additional tools, training or support regarding PSS registration and classification. Still, while GPs provide several suggestions for improvements of the classification system, there is little consensus on this matter.

Looking more specifically at the first aim of this study, many GPs are struggling with registration of PSS and are hesitant to use codes beyond the somatic complaints they objectively observe. This is in line with instructions of the ICPC [13, 28] and previous research findings, reporting that GPs’ fear of stigmatization may lead them to avoid codes related to social and psychological problems [29, 36, 37]. On the other hand, respondents did indicate more frequently diagnosing the PSS-related syndrome IBS, compared to CFS and FM, which is in line with previous research indicating that GPs are more proficient in diagnosing IBS [11]. This suggests that registration behaviour may be more depended upon the GP’s confidence in classifying PSS than upon fear of stigmatization.

Regarding the second aim of this study, our results show that the current registration and classification options for PSS are insufficient for a substantial number of GPs. These GPs reportedly require a specific code for PSS, in combination with training, tools, a widely accepted guideline, and referral options. In contrast, the literature shows that there are a variety of training options [38], concise and validated screening questionnaires [26, 39, 40], and referral options [3, 13, 41] available to GPs. Besides, the Dutch GP association has an elaborate PSS guideline [13]. In line with this guideline, some GPs suggested coding of PSS should be done by severity, which is also in line with studies which propose the introduction of codes that specify severity to improve documentation of mild PSS [8, 9, 29]. Interestingly, research demonstrated that the GPs’ use of subcategories directed at classifying severity is challenged by the GPs’ conceptual understanding of PSS [8]. It is therefore conceivable that GPs do indeed need training, and knowledge of the availability of training, to increase their understanding of PSS.

### Strengths and limitations

To the best of our knowledge, this study is the first to capture an overview of GPs’ perspectives regarding registration and classification of PSS through exploration of their specific ICPC-registration behaviour. Our data sheds light on GPs’ reasoning regarding PSS, confirms the lack of consensus on registration and classification and offers guidance for improvements in registration and classification based on the GPs’ reported needs. Nonetheless, some limitations should be noted. First, in order to distribute the survey as broadly as we have, we involved third parties (i.e. regional GP-networks) to promote distribution. This resulted in a limited overview of the number of GPs reached, leading to a rough estimate of the response rate. Second, responses may have been biased by elective participation. Still, although adequate reference data is limited, responses appear fairly representative for the population of GPs in the Netherlands [35, 42]. Regarding the content of the survey, a strength is that face valid answers were facilitated for the choice in ICPC codes by presenting GPs with codes in a drop-down menu, similar to their EHRs’ set-up. Nonetheless, this came with the limitation that it is unclear if the more frequently coded ‘P75-somatization disorder’ in case of fatigue compared to other complaints is a true finding, or whether it demonstrates the limitations of the ICPC coding system itself to facilitate classification of PSS, or if it is related to a lack of potential alternative codes (see additional file 2). Lastly, generalisation of the findings should be done with caution, since many questions were based on hypothetical situations.

### Practical implications

The great variance in responses and methods for registration of PSS found in our research suggests that clinical practice may be improved by better registration of PSS. Improving classification and providing adequate registration options may support GPs in the overall care for PSS. To improve registration, a clear definition with a specific code for PSS should be implemented in the ICPC system. Introduction of such a code should be combined with (communication on) training options for GPs, which also broadens the GPs’ knowledge on currently available diagnostic tools, guidelines and referral options. Besides providing more accessible coding and training options, research could support the GP further by developing a data-based screening tool for early identification of patients at risk for PSS. This could be another way to support the GP with their challenges in conceptualizing PSS. Besides, this may promote timely treatment of the cognitive, emotional and behavioural components of PSS, which, in turn, may decrease the burden of PSS and reduce the risk of iatrogenic harm.

## Conclusion

Registration of PSS in primary care is currently ambiguous. Specific complaints presented by patients with PSS are primarily coded on a symptom-specific level. Approximately half of GPs expressed a need for more coding options for PSS and over half of GPs reported a need for further training, tools or other support regarding PSS. Since many of the latter already exist, improvements should be directed at new options for registration, specifically coding, and increasing and spreading knowledge about PSS, guidelines, available tools and referral options.

## Supplementary Information

**Additional file 1**. Survey translation: SOLK in general practice, a general practitioners’ perspective. Contains all survey questions and definitions outlined in the measures section.

**Additional file 2**. Overview of items, ICPC codes and categorization. Contains a list of ICPC codes presented to the general practitioners participating in the present study. Also including categorization according to the complaint the patient presented with and the category according to type of ICPC code.

**Additional file 3**. Registration of PSS-related complaints using ICPC. Contains a table which summarizes the results of the first survey question regarding the ICPC codes general practitioners are most likely to use when a patient presents with a specific PSS-related complaint. The table gives a more detailed description of what is depicted in Fig. 1.

## Data Availability

The datasets generated and/or analysed during the current study are not publicly available due to lack of consent of participants but are available from the corresponding author upon reasonable request.

## Abbreviations

PSS: Persistent somatic Symptoms
GP: General Practitioner
EHR: Electronic health records
ICPC: International Classification of Primary Care
IBS: Irritable bowel syndrome
CFS: Chronic fatigue syndrome
FM: Fibromyalgia
MUPS: Medically unexplained physical symptoms
SSD: Somatic symptom disorder
SOLK: Somatisch onvoldoende verklaarde lichamelijke klachten (Dutch translation of MUPS
