# Correction to: The general practitioners perspective regarding registration of persistent somatic symptoms in primary care: a survey

**DOI:** 10.1186/s12875-021-01566-x

**Published:** 2021-11-23

**Authors:** Willeke M. Kitselaar, Rosalie van der Vaart, Madelon van Tilborg-den Boeft, Hedwig M. M. Vos, Mattijs E. Numans, Andrea W. M. Evers

**Affiliations:** 1 Health, Medical and Neuropsychology Department, Leiden University, Faculty of Social and Behavioral Sciences, Leiden, the Netherlands; 2 Public Health and Primary Care Department / LUMC-Campus Den Haag, Leiden University, Medical Center, The Hague, the Netherlands

**Correction to: BMC Fam Pract 22, 182 (2021)**

10.1186/s12875-021-01525-6

In the original publication of this article [1], Fig. 1 image was incorrect, the correct is shown in the following page. Also, there was a typographical error for the word “ambiguous” under Background section in the Abstract which was written as “unambiguous”.

**Fig. 1:**
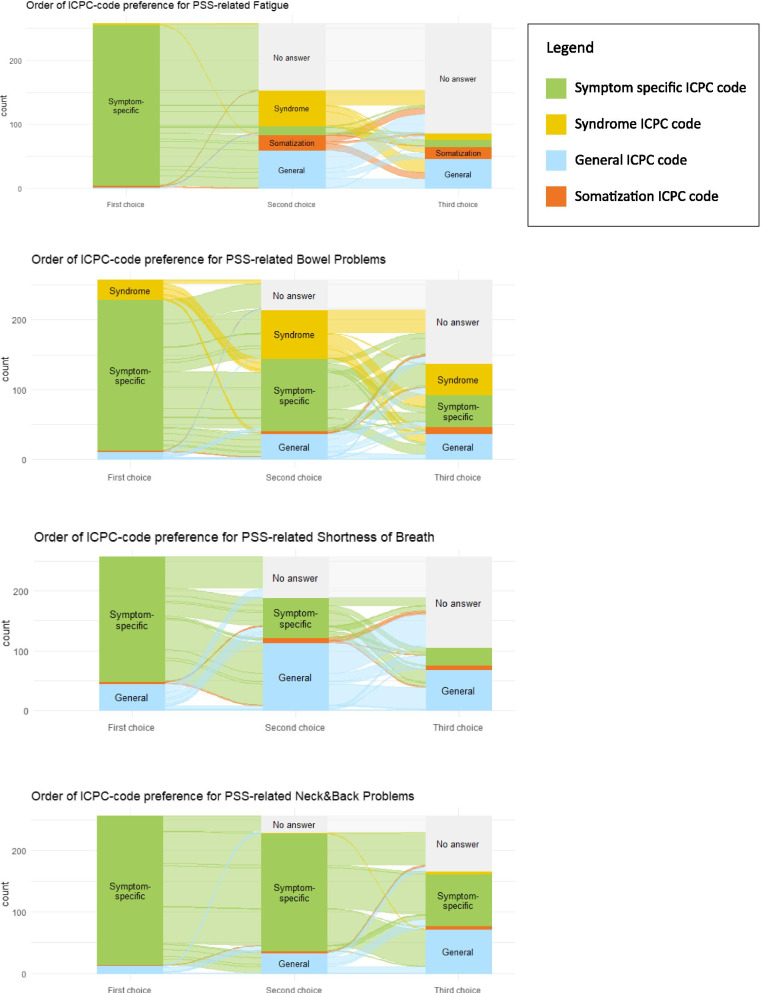
Visualizations of general practitioners’ order of choosing ICPC-codes for specific persistent somatic symptoms (PSS)

The original article has been corrected.
