# Supplementary material for: The general practitioners perspective regarding registration of persistent somatic symptoms in primary care: a survey
Source: BMC Fam Pract. 2021 Sep 11;22:182. doi: 10.1186/s12875-021-01525-6 (PMC8436507; doi:10.1186/s12875-021-01525-6)
Supplement: Supplementary file 1 — Additional file 1. Survey translation: SOLK in general practice, a general practitioners’ perspective. Contains all survey questions and definitions outlined in the measures section. [file 12875_2021_1525_MOESM1_ESM.pdf]

SURVEY TRANSLATION: SOLK in general practice, a general practitioners' perspective

This file contains SOLK-related definitions and questions outlined in the measures section of the article are presented below.

Definition of 'somatisch onvoldoende verklaarde lichamelijke klachten' (SOLK):
We speak of SOLK when regular medical care cannot find an adequate explanation for the complaints with which the patient presents him/herself. Patients with a well-known somatic condition can also have SOLK, either presenting with a totally different complaint or presenting with more severe complaints regarding their diagnosed condition than would be expected.

Part 1: *Imagine a patient visiting your office who has consulted you frequently in the precious 6 months with the same or differing complaints. Extensive research has excluded a medical explanation for the complaint(s).*
For each complaint presented, choose the ICPC-code which you would use most often. You can choose a maximum of three ICPC-codes per complaint.

a) Bowel problems

Three drop-down menus (see additional file 2 for list of ICPC codes)

b) Fatigue

Three drop-down menus (see additional file 2 for list of ICPC codes)

c) Neck and back pain

Three drop-down menus (see additional file 2 for list of ICPC codes)

d) Shortness of breath

Three drop-down menus (see additional file 2 for list of ICPC codes)

(Each complaint was presented on a new page with the description of the hypothetical patients at the top of the page)

Part 2: Do you use the ICPC-codes for fibromyalgia (L18.01), chronic fatigue syndrome (A04.01) and/or irritable bowel syndrome (D93)? (multiple answers possible)

- ☐ Yes, I diagnose these syndromes myself sometimes
- ☐ Yes, I use these codes when the diagnosis is made by a medical specialist
- ☐ No, I prefer reporting these complaints at a symptom level
- ☐ No, I am not convinced that these are discernable syndromes
- ☐ Other, namely…

Comments: _

Part 3: The next questions are about if or how you report SOLK in your electronic health record (EHR) when you have enough reasons to assume that the complaints are SOLK.

	never	now and then	often	always
a) Do you write SOLK in the episode name?	○	○	○	○
b) Do you write SOLK in the free text area of the EHR?	○	○	○	○

Comments: _

c) There is no unambiguous way to categorize severe SOLK in the EHR (there is no ICPC-code for SOLK).
Is this a problem for you?

○ No

○ Yes

Comments: _

Part 4: The questions below are about registration of SOLK in case on a (co-morbid) explained medical condition.
Imagine a patient with a well-known medically explained disorder (such as, Crohn's disease or asthma) visiting your office. This patient experiences the disease differently than most people with this disorder; this patient may have disproportion thoughts, feelings or behaviors related to the disorder or displays severe disruptions in daily functioning compared to other patients who have the disorder in similar severity.

a) Would you report this in your EHR?

○ No

○ Yes

Comments: _

b) Would you like to have the option to report this (behavior) in a specific ICPC-code?

○ No

○ Yes

Comments: _

Part 6: Research indicates that (primary care) physicians often experience difficulties with consultations and treatment of patients with SOLK. The following questions are addressing this.

a) Do you have a need for an (online) tool for SOLK?

- ○ No
- ○ Yes, namely…

Comments: ___________________________________

b) Do you have a need for training regarding SOLK?

- ○ No
- ○ Yes, namely…

Comments: ___________________________________

c) Do you have a need other support regarding care for patients with SOLK?

- ○ No
- ○ Yes, namely…

Comments: ___________________________________
