# Supplementary material for: The general practitioners perspective regarding registration of persistent somatic symptoms in primary care: a survey
Source: BMC Fam Pract. 2021 Sep 11;22:182. doi: 10.1186/s12875-021-01525-6 (PMC8436507; doi:10.1186/s12875-021-01525-6)
Supplement: Supplementary file 2 — Additional file 2. Overview of items, ICPC codes and categorization. Contains a list of ICPC codes presented to the general practitioners participating in the present study. Also including categorization according to the complaint the patient presented with and the category according to type of ICPC code. [file 12875_2021_1525_MOESM2_ESM.pdf]

Overview of items, ICPC codes and categorization

		Type of complaint				
**ICPC**	**Name**	**Neck & back**	**Bowel**	**Shortness of breath**	**Fatigue**	**Category**
**A01**	Pain general/multiple sites	X	X			General
**A04**	Weakness/tiredness general			X	X	Symptom-specific
**A04.01**	Chronic fatigue syndrome			X	X	Syndrome
**A28**	Limited function/disability NOS	X	X	X	X	General
**A97**	No disease	X	X	X	X	General
**A99**	General disease NOS	X	X	X	X	General
**D01**	Abdominal pain/cramps general		X			Symptom-specific
**D02**	Abdominal pain epigastric		X			Symptom-specific
**D06**	Abdominal pain localized other		X			Symptom-specific
**D20**	Mouth/tongue/lip symptom/complaint			X		Symptom-specific
**D28**	Limited function/disability (d)		X			General
**D29**	Digestive symptom/complaint other		X			General
**D93**	Irritable bowel syndrome		X			Syndrome
**K02**	Pressure/tightness of heart			X		Symptom-specific
**K03**	Cardiovascular pain NOS			X		General
**K04**	Palpitations/awareness of heart			X		Symptom-specific
**K05**	Irregular heartbeat other			X		Symptom-specific
**L01**	Neck symptom/complaint	X				Symptom-specific
**L02**	Back symptom/complaint	X				Symptom-specific
**L03**	Low back symptom/complaint	X				Symptom-specific
**L18**	Muscle pain	X				Symptom-specific
**L18.01**	Fibromyalgia	X				Syndrome
**L27**	Fear musculoskeletal disease other	X				General
**L28**	Limited function/disability (l)	X				General
**L29**	Sympt./complaint musculoskeletal other	X				General
**L79**	Sprain/strain of joint NOS	X				Symptom-specific
**L79.01**	Whiplash trauma cervical spine	X				Symptom-specific
**L83**	Neck syndrome	X				Symptom-specific
**L86**	Back syndrome with radiating pain	X				Symptom-specific
**L86.01**	Spinal disc herniation (thoracic/lumbar)	X				Symptom-specific
**N01**	Headache	X				Symptom-specific
**N28**	Limited function/disability (n)	X	X			General
**N29**	Neurological symptom/complaint other	X	X	X		General
**P28**	Limited function/disability (p)	X	X	X	X	General
**P29**	Psychological symptom/complaint other	X	X	X	X	General
**P75**	Somatization disorder	X	X	X	X	Somatization
**P99**	Psychological disorders, other	X	X	X	X	General
**R01**	Pain respiratory system			X		Symptom-specific
**R02**	Shortness of breath/dyspnoea			X		Symptom-specific
**R04**	Breathing problem, other			X		General
**R28**	Limited function/disability (r)			x		General
**U13**	Bladder symptom/complaint other		X			General
**U28**	Limited function/disability urinary		X			General
