# Supplementary material for: The general practitioners perspective regarding registration of persistent somatic symptoms in primary care: a survey
Source: BMC Fam Pract. 2021 Sep 11;22:182. doi: 10.1186/s12875-021-01525-6 (PMC8436507; doi:10.1186/s12875-021-01525-6)
Supplement: Supplementary file 3 — Additional file 3. Registration of PSS-related complaints using ICPC. Contains a table which summarizes the results of the first survey question regarding the ICPC codes general practitioners are most likely to use when a patient presents with a specific PSS-related complaint. The table gives a more detailed description of what is depicted in Fig. 1. [file 12875_2021_1525_MOESM3_ESM.pdf]

Registration of PSS-related complaints using ICPC (n = 259)

Registration method	First choice [a]	Second choice [a]	Third choice [a]
**Combination [b]**	**n = 1035**	**n = 791**	**n = 500**
Symptom-specific [c]	924 (89.3)	377 (47.7)	172 (34.4)
General [d]	71 (6.9)	246 (31.1)	227 (45.5)
Somatization [e]	10 (1.0)	40 (5.1)	40 (8.0)
Syndrome [f]	30 (2.9)	128 (16.1)	61 (12.2)
**Fatigue**	**n = 259**	**n = 154**	**n = 88**
Symptom-specific [c]	252 (97.3)	14 (9.1)	11 (12.5)
General [d]	2 (0.8)	61 (39.6)	47 (53.4)
Somatization [e]	3 (1.2)	24 (15.6)	18 (20.5)
Syndrome [f]	2 (0.8)	55 (35.7)	12 (13.6)
**Bowel problems**	**n = 259**	**n = 216**	**n = 139**
Symptom-specific [c]	218 (84.2)	104 (48.1)	46 (33.1)
General [d]	11 (4.2)	37 (17.1)	38 (27.3)
Somatization [e]	2 (0.8)	4 (1.9)	10 (7.2)
Syndrome [f]	28 (10.8)	71 (32.9)	45 (32.4)
**Shortness of Breath**	**n = 259**	**n = 190**	**n = 107**
Symptom-specific [c]	210 (81.1)	67 (35.3)	30 (28.0)
General [d]	45 (17.4)	114 (60.0)	70 (65.4)
Somatization [e]	4 (1.5)	9 (4.7)	7 (6.5)
Syndrome [f]	0 (0)	0 (0)	0 (0)
**Neck and back pain**	**n = 258**	**n = 231**	**n = 166**
Symptom-specific [c]	244 (94.6)	192 (83.1)	85 (51.2)
General [d]	13 (5.0)	34 (14.7)	72 (43.4)
Somatization [e]	1 (0.4)	3 (1.3)	5 (3.0)
Syndrome [f]	0 (0)	2 (0.9)	4 (2.4)

[a] Top three ICPC codes (by category) GPs most likely choose for PSS-related complaints (second and third choice elective).

[b] Combination of all responses for the four complaints (fatigue, bowel problems, shortness of breath, and neck and back pain).

[c] Includes range of (mainly symptomatic) ICPC codes.

[d] Includes range of more general ICPC codes without specified location or diagnosis.

[e] P75 – somatization disorder.

[f] Fatigue: A04.01 – CFS; bowel problems: D93 – IBS; shortness of breath: no code available; neck and back pain: L18.01 - FM.
